# T lymphocytes export proteasomes by way of microparticles: a possible mechanism for generation of extracellular proteasomes

**DOI:** 10.1111/jcmm.12160

**Published:** 2013-10-31

**Authors:** Isabel Bochmann, Frédéric Ebstein, Andrea Lehmann, Jeremias Wohlschlaeger, Stephan Urs Sixt, Peter-Michael Kloetzel, Burkhardt Dahlmann

**Affiliations:** a Institut für Biochemie, Charité-Universitätsmedizin BerlinBerlin, Germany; b Institut für Pathologie and Neuropathologie, Universität Duisburg-EssenEssen, Germany; c Klinik für Anaesthesiologie, Heinrich-Heine-Universität DüsseldorfDüsseldorf, Germany

**Keywords:** proteasome, circulating, extracellular, microparticles, sphingomyelinase, T lymphocytes

## Abstract

The 20S proteasome is almost exclusively localized within cells. High levels of extracellular proteasomes are also found circulating in the blood plasma of patients suffering from a variety of inflammatory, autoimmune and neoplastic diseases. However, the origin of these proteasomes remained enigmatic. Since the proteome of microparticles, small membrane enclosed vesicles released from cells, was shown to contain proteasomal subunits, we studied whether intact proteasomes are actively released into the extracellular space. Using human primary T lymphocytes stimulated with CaCl_2_ and the calcium ionophore A23187 to induce membrane blebbing we demonstrate that microparticles contain proteolytically active 20S proteasomes as well as the proteasome activator PA28 and subunits of the 19S proteasome regulator. Furthermore, our experiments reveal that incubation of *in vitro* generated T lymphocyte-microparticles with sphingomyelinase results in the hydrolysis of the microparticle membranes and subsequent release of proteasomes from the vesicles. Thus, we here show for the first time that functional proteasomes can be exported from activated immune cells by way of microparticles, the dissolution of which may finally lead to the generation of extracellular proteasomes.

## Introduction

Extracellular proteasomes circulating in blood plasma (circulating proteasomes) are potential diagnostic biomarkers for various malignancies like systemic lupus erythematodes (SLE) 1, melanoma 2, multiple myeloma 3, epithelial ovarian cancer 4 and hepatocellular carcinoma 5–6. Clear correlations were found to exist between the plasma level of extracellular proteasomes and the malignant transformation of cirrhotic liver cells 6 as well as residual tumour mass after therapy in patients suffering from epithelial ovarian cancer 4.

The proteasome catalyses the degradation of intracellular proteins and is involved in vital functions like cell cycle progression, differentiation, immune defence and the stress response 7–8. The barrel-shaped 20S proteasome is composed of four stacked, seven-membered rings, two inner β and two outer α rings. The α rings provide contact to 19S regulators or to the proteasome activator PA28 thus forming 26S proteasomes (19S-20S), 30S proteasomes (19S-20S-19S), PA28-20S proteasome-complexes or hybrid proteasomes (PA28-20S-19S), respectively. The β rings contain the three proteolytically active subunits with β1 exhibiting caspase-like, β2 trypsin-like and β5 chymotrypsin-like activity. Cytokines like interferon-γ induce the expression of the immuno-subunits β1i (LMP2), β2i (MECL1) and β5i (LMP7), which replace the standard-subunits β1, β2 and β5 resulting in the biogenesis of immuno-proteasomes 7–8. Immunoproteasomes are constitutively generated by cells involved in the immune response like spleenocytes, thymocytes, dendritic cells and others 9.

Circulating proteasomes from human plasma are proteolytically active 20S proteasomes 10. They were also detected in the lung epithelial lining fluid of patients with acute respiratory distress syndrome 11 and in cerebrospinal fluid 12. Apart from their importance as diagnostic biomarkers extracellular proteasomes may also exert various extracellular functions 13–14. However, so far the mechanisms by which proteasomes are released into the extracellular space remained obscure 15. Although a passive efflux of proteasomes as a result of cell death occurring in the course of various diseases cannot be excluded, several observations argue in favour of a specifically directed mechanism 13–15.

Proteasomal subunits contain no signal sequence for export *via* the classic secretory pathway. Therefore, alternative secretion mechanisms might be responsible for proteasome release to the extracellular environment 16,17, *e.g*. secretory lysosomes and plasma-membrane-residing transporters. But so far there exists no evidence for a proteasome-interacting transporter at the plasma membrane. Another alternative export pathway releases exosomes, which are 50–100 nm vesicular structures. Finally, a membrane blebbing pathway was described whereby cytoplasmic content is entrapped in protrusions of the membrane, which are then directly shed as vesicles (microparticles) of heterogeneous size (0.1–1 μm) from the plasma membrane of activated cells. During the last decade evidence was provided that microparticles (MP) can act as messengers between cells since they transport a variety of molecules 19–22. Proteomic analyses of microparticles derived from platelets and a T lymphocyte cell line have revealed the presence of subunits of the proteasome, the proteasome activator PA28 and the ubiquitination machinery 23–24. By means of immunoelectron microscopy proteasomes were also identified in vesicles in cerebrospinal fluid 12. Therefore, we investigated whether membrane blebbing and shedding of vesicles as well as their subsequent breakdown may be a mechanism by which proteasomes are released from cells thereby generating extracellular proteasomes.

## Materials and methods

### Materials

Roswell Park Memorial Institute (RPMI) 1640 medium without phenolred, foetal calf serum (FCS), penicillin/streptomycin, L-glutamine and biocoll reagent were from Biochrom AG (Berlin, Germany). A23187, ethylene glycol tetraacetic acid (EGTA) and sphingomyelinase were purchased from Sigma-Aldrich (Taufkirchen, Germany). Epoxomicin was from Calbiochem (Nottingham, UK). The polyclonal proteasome antibody (PW8155), the monoclonal α6- and β1- and the polyclonal β5-antibodies were from Enzo Life Sciences (Lörrach, Germany). The polyclonal antibodies for β1i/LMP2 and β5i/LMP7 and the monoclonal LFA-1-antibody were from Abcam (Cambridge, UK). The Rpt3 antibody was a kind gift from Klavs Hendil (Copenhagen, Denmark). The polyclonal rabbit PA28α antibody was a laboratory stock. The PA28β antibody was from Cell Signalling (Frankfurt, Germany). 20S and 26S proteasome as well as PA28 were prepared from human erythrocytes 25 and 20S proteasome from spleenocytes were from BioMol (Exeter, UK). The Trucount® tubes, CD3-PE antibody and annexin V-FITC were from BD Biosciences (Heidelberg, Germany). CytoTox One homogenous membrane integrity assay was from Promega (Mannheim, Germany).

### Cell culture

T lymphocytes were isolated from buffy coats of healthy donors provided by German Red Cross (Berlin, Germany). They were maintained in RPMI 1640 medium without phenolred supplemented with 10% (v/v) heat-inactivated FCS, 100 U/100 μg/ml penicillin/streptomycin and 2 mM L-glutamine (complete medium) at 37°C in a 5% CO_2_ atmosphere up to 1 week after isolation. For the experiments 5 × 10^7^ cells were centrifuged (300 × *g*, 8 min.) and taken up in 6 ml of serum-free or microparticle-free (MP-free) medium. MP-free medium is complete medium supplemented with FCS centrifuged at 100,000 × *g* over night to remove bovine plasma microparticles.

### Isolation of T lymphocytes

Buffy coats were diluted with 0.5–1 volume of RPMI 1640 medium (without supplements) and 40 ml of the cell suspension were covered on 12 ml Biocoll reagent and centrifuged for 30 min. at 20°C (675 × *g*). Peripheral blood mononuclear cells (PBMCs) remaining in the layer above the Biocoll reagent were isolated and washed three times in RPMI (8 min., 300 × *g*, 4°C). For separation of monocytes from T and B cells, PBMCs were diluted in RPMI and incubated in cell culture flasks for 3–4 hrs at 37°C in a 5% CO_2_ atmosphere. Under these conditions monocytes adhere to the cell culture flask and the supernatant contains the T and B lymphocytes. 85% of the isolated cells were CD3-positive T cells as analysed by flow cytometry (data not shown).

### Preparation of microparticles

5 × 10^7^ T cells were stimulated with 2 μM A23187 calcium ionophore in serum-free or MP-free medium supplemented with 1 mM CaCl_2_ 26. Control cells were stimulated with DMSO or DMSO plus 1 mM CaCl_2_ at 37°C in a 5% CO_2_ atmosphere for 45 min., respectively_._ The medium containing cells and microparticles was subjected to differential centrifugation at 470 × *g* for 5 min. at 4°C to remove the cells. The supernatant was transferred to a second centrifugation (1400 × *g*, 10 min., 4°C) to remove larger debris. To isolate microparticles, the supernatant was centrifuged at 100,000 × *g* for 20 min. at 4°C. The centrifugation step was repeated two times if washing of the microparticles with PBS was required. The effect of Ca^2+^/A23187 treatment on cell viability was checked by trypan blue staining and LDH release (CytoTox ONE) according to the manufacturer’s protocol.

### Quantification of 20S proteasome

Quantification of 20S proteasome was carried out by ELISA after breakdown of the microparticles in PBS supplemented with 1% Tween 20 by repeated freeze-thaw cycles and subsequent centrifugation to remove residual lipids (45 min., 20,000 × *g*, 4°C). The capturing antibody against the proteasome-subunit α6, dissolved 1 μg/ml PBS, was bound to an ELISA plate over night at 4°C. After washing with PBST (PBS containing 0.2% Tween 20), the plate was blocked with 1% albumin fraction V dissolved in PBST for 2 hrs and the washing step was repeated. On each ELISA plate purified erythrocyte 20S proteasome was used as calibration standard. The samples (plasma samples were diluted 1:2) and proteasome standards were detected with a polyclonal antibody to 20S proteasome (dilution 1:1000), the binding of which was photometrically quantified with a peroxidase-coupled rabbit IgG antibody and TMB substrate (BD Biosciences). The detection range was 2–60 ng of proteasome.

### Proteasome activity

The proteasome activity was measured by cleavage of fluorogenic peptide-substrates as described elsewhere 10. T cells or proteasome-containing microparticles and substrates were resuspended or dissolved in TEAD buffer (20 mM Tris-HCl, 1 mM EDTA, 1 mM NaN_3_, 1 mM dithiothreitol; pH 7.5). For detection of the different proteasomal activities fluorogenic peptide substrates Suc-LLVY-AMC (chymotrypsin-like activity), Bz-VGR-AMC (trypsin-like activity) and Z-LLE-AMC (caspase-like activity) (Bachem, Bubendorf, Switzerland) were used at final concentrations of 100, 200 and 200 μM, respectively. Proteasome inhibition was performed with 1 μM epoxomicin.

### Flow cytometric detection and quantification of microparticles

Microparticles were generated and isolated as described and washed two times in sterile filtered PBS. After the last centrifugation step, the microparticles were suspended in 200 μl sterile-filtered annexin V binding buffer, transferred to Trucount® tubes and stained with FITC-annexin V. After 15 min. of incubation the samples were diluted by 400 μl annexin V binding buffer and directly analysed with an FACS Calibur flow cytometer (BD Biosciences). The size analysis with Megamix beads was accomplished according to the manufacturer′s protocol (Biocytex, Marseille, France).

### Western blot analysis

T cells (5 × 10^7^) were stimulated with 1 mM CaCl_2_ and 2 μM A23187 calcium ionophore in serum-free medium for 45 min. before cells and microparticles were separated by centrifugation. The pelleted cells were washed with PBS and resuspended in 100 μl of RIPA buffer (10 mM Tris-HCl pH 7.5, 100 mM NaCl, 1% Triton X-100, 1% sodium deoxycholate, 0.1% SDS) supplemented with Complete protease inhibitor mix (Roche, Basel, Switzerland); the microparticles were suspended in 20–40 μl RIPA buffer. Cells and microvesicles were then disrupted by repeated freeze-thaw cycles and undissolved material precipitated at 20,000 × *g* for 40 min. at 4°C. The microparticle supernatants were mixed with sample buffer (20 mM Tris-HCl pH 6.8, 10% (w/v) glycerol, 5% β-mercaptoethanol, 2% SDS, 0.05% bromphenol blue) and entirely loaded on the SDS-gel. Protein concentrations were measured in cell lysate by means of BCA assay and equal amounts of protein were subjected to SDS-PAGE (12% w/v). The proteins were immunoblotted and detected by using the ECl Reagent.

### Digestion of microparticles with sphingomyelinase

For enzymatic breakdown of microparticles, the isolated microvesicles were resuspended in 10 mM HEPES, pH 7.4, containing 140 mM NaCl, 2.7 mM KCl, 1 mM MgCl_2_, 5 mM glucose and 1 mM CaCl_2_ and then incubated at 37°C with or without 0.1 U/ml sphingomyelinase from *Bacillus cereus* 26. After 1 hr of enzymatic digestion remaining vesicle residues were pelleted at 20,000 × *g* for 40 min. and the supernatants analysed for 20S proteasomes by ELISA. The membrane fraction was resuspended in RIPA buffer supplemented with complete protease inhibitor cocktail, the membranes were disrupted by repeated freeze-thaw cycles and after centrifugation the supernatant was subjected to SDS-PAGE.

### Native PAGE

Microparticles were isolated from 2.5 × 10^8^ T lymphocytes and suspended in a 10:1 (v/v) mixture of TSDG buffer (10 mM Tris-HCl, 25 mM KCl, 10 mM NaCl, 1.1 mM MgCl_2_, 0.1 mM EDTA, 1 mM DTT, 2 mM ATP, 10% glycerol, pH 7.2) and native gel loading buffer (5 mM Tris-HCl, 5% glycerol and 0.01% bromphenolblue). Electrophoresis was performed on 3–12% Native PAGE BisTris gels before the proteasomal trypsin-like activity was visualized by substrate overlay with 200 μM Bz-VGR-AMC. Alternatively the proteasomes were analysed by immunoblotting.

### Blood collection and microparticle isolation of human plasma

Twenty millilitre peripheral blood was collected from healthy donors with 21-gauge needles in Vacutainer tubes (BD Biosciences) supplemented with citrate. Platelet-free plasma was produced by different centrifugation steps, 500 × *g* for 10 min and two times at 2000 × *g* for 30 min. 27. One millilitre platelet-free plasma was diluted 1:4 (v/v) with PBS and microparticles were isolated at 100,000 × *g* for 40 min. at 4°C, washed with PBS and collected at 100,000 × *g* for 20 min. The supernatant fraction was used for the quantification of free plasma proteasomes and the pelleted fraction for vesicle-bound proteasomes. The latter were also analysed by immuno blotting.

### Statistical analysis

Statistical comparison of two groups of data was done by Student’s *t*-test or in case of equal variance by Mann–Whitney Rank Sum Test. Error bars display standard error of the mean (±SEM) values. *P*-values lower than 0.05 were considered statistically significant.

## Results

### Cellular and vesicular T lymphocyte proteasomes

**Figure 1:**
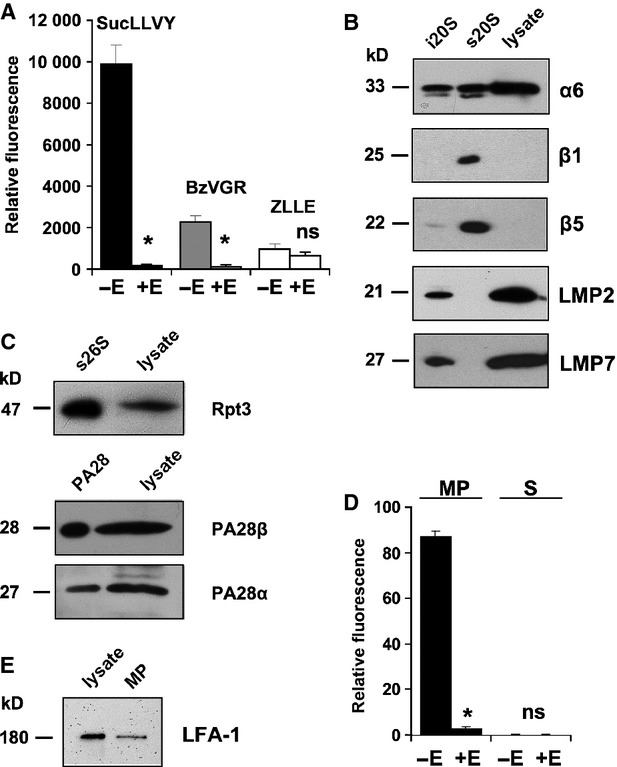
Analysis of proteasomal activity and subunit composition in T lymphocytes. T lymphocytes were isolated from buffy coats of healthy donors. (A) T lymphocyte lysates were tested for proteasomal activity with the substrates Suc-LLVY-AMC (black columns), Bz-VGR-AMC (grey columns) and Z-LLE-AMC (white columns) in the presence and absence of 1 μM epoxomicin (±E). Means ± SEM (*n* = 3) are shown and *P*-values −E *versus* +E were calculated (*P ≤ 0.002; ns: not significant). (B) 20 ng immunoproteasomes (i20S), 20 ng standard proteasomes (s20S) and 20 μg T cell lysates were subjected to SDS-PAGE and analysed by immunoblotting for their content of the proteasomal subunits α6, β1, β5, LMP2 and LMP7, respectively. Representative blots of three independent experiments are shown. (C) Detection of the 19S subunit Rpt3 and proteasome activator PA28 subunits α and β in T cell lysate. 26S proteasome (1 μg) and PA28 (400 ng) purified from human erythrocytes were used as controls. (D) Three hours after exchange of the cell culture medium by microparticle-free medium proteasomal activity was measured in the cell culture supernatant (S) and in the microparticle fraction (MP) with Suc-LLVY-AMC in the presence (+E) and absence (−E) of 1 μM epoxomicin. Means ± SEM (*n* = 3) are given (*P ≤ 0.001; ns: not significant). (E) Detection of the T lymphocyte surface protein LFA-1 in T cell lysates and vesicle fraction.

Microparticles of T lymphocytes and other blood cells were found in blood plasma of healthy individuals and patients suffering from various diseases 28. We hypothesized that microvesicles may be a source for extracellular proteasomes. To allow comparison of T cell derived proteasomes with those that may be present in T cell derived microvesicles we initially analysed proteasomes in primary T lymphocytes of healthy donors. T cell lysates incubated with proteasome-specific fluorogenic peptide substrates showed all three proteasomal activities. The chymotrypsin- and trypsin-like activities could be inhibited by epoxomycin (*P* ≤ 0.001 and 0.002, respectively, Fig. 1A). To investigate, which type of proteasome is present in T lymphocytes, lysates of T cells were subjected to SDS-PAGE and analysed for standard- (β1, β2 and β5) and immuno-subunits β1i (LMP2), β5i (LMP7). T cell lysates showed signals solely of immuno-subunits LMP2 and LMP7 (Fig. 1B) indicating that exclusively immunoproteasomes are constitutively present in these cells. In addition, the T cell lysates contained components of the 19S regulatory particle (Rpt3) and the proteasome activator PA28 (PA28α and β) (Fig. 1C). To explore whether the culture medium of T lymphocytes contains extracellular proteasomes the culture medium was subjected to differential centrifugation to separate remaining cells and debris. Thereafter, the cell-free medium was ultracentrifuged to precipitate microvesicles and to obtain a vesicle-free culture supernatant. Both the supernatant and the vesicle fraction were incubated with the membrane-permeable substrate Suc-LLVY-AMC to measure proteasomal chymotrypsin-like activity. As shown in Figure 1D proteasomal activity was measured only in the microvesicle fraction indicating that extracellular proteasomes in the cell culture medium are associated with vesicular structures.

### Characterization of microparticles shed by activated T lymphocytes

If vesicle shedding by T cells results from cell membrane blebbing, microvesicles are expected to contain proteins originating from their parental cell 28. Therefore, the vesicular membranes were dissolved by detergents and tested for the presence of a T cell membrane-localized protein, the leucocyte-derived integrine lymphocyte-function associated antigen-1 (LFA-1). Figure 1E clearly revealed the existence of LFA-1 in the vesicle membranes suggesting that membrane blebbing and shedding occurs continuously.

**Figure 2:**
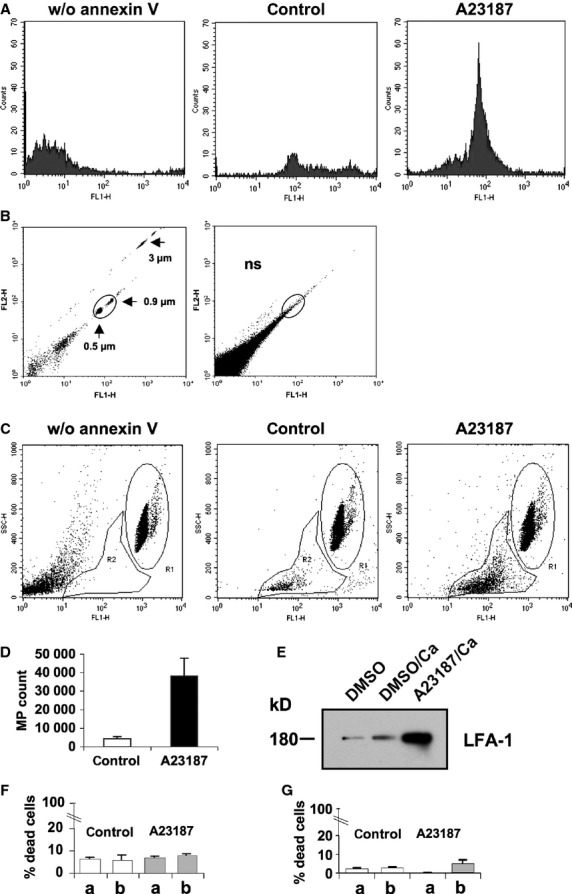
Flow cytometric analysis of isolated microparticles. Microparticle (MP) fractions of T lymphocytes incubated with DMSO (control) and MP fractions of cells stimulated for 45 min with 2 μM A23187 and 1 mM CaCl_2_ (A23187) were labelled with annexin V-FITC. (A) Detection of annexin V-labelled T cell microparticles by flow cytometry. (B) Dot plots of Megamix beads (0.5, 0.9 and 3 μm) and annexin V-stained MP of T cells stimulated with 2 μM A23187 and 1 mM CaCl_2_ for size analysis in the MP gate. (C) Quantification of MP derived from stimulated (A23187) and unstimulated T cells (control). The relative granularity SSC was measured with FACS Calibur. For absolute quantification, 5000 Trucount beads were counted in region 1 (R1) and the annexin V-positive MP were counted in region 2 (R2). (D) Means ± SEM of the analysis of experiments as described in (C) are given and statistically compared with the Mann–Whitney Rank Sum test (*P ≤ 0.001). (E) A representative immunoblot for detection of LFA-1 in the total vesicle fraction of DMSO, DMSO/1 mM CaCl_2_ and 1 mM CaCl_2_/2 μM A23187 treated T cells, respectively, is shown. (F) Aliquots of 3.5 × 10^6^ T lymphocytes were stained with trypan blue before (a) and after (b) treatment with DMSO/1 mM CaCl_2_ (control; *P* = 0.968) and A23187/CaCl_2_ (A23187; *P* = 0.149) and then counted. (G) Similarly as described in (F) live and dead cells were measured by LDH release (control, *P* = 0.232; A23187, *P* = 0.4).

From earlier studies it is known that cells can be activated by the A23187 calcium ionophore and CaCl_2_ resulting in an enhanced segregation of microparticles from the plasma membrane 26. Therefore, we stimulated T lymphocytes with A23187 and CaCl_2_ before the vesicle fraction was isolated and analysed by flow cytometry (Fig. 2). During the shedding process, the parental cells lose their membrane symmetry at vesicle exit sites. This leads to exposure of phosphatidylserine residues to the outer leaflet of the plasma membrane 28 thereby allowing the specific binding of annexin V to phosphatidylserine. Together with the particle size this feature can serve as a marker to differentiate microparticles from other particulate structures 29. As shown in Figure 2A flow cytometric analysis of the vesicles derived from the T cell fraction revealed binding of annexin V-FITC and this signal was strongly enhanced after stimulation of vesicle blebbing. A comparative dot plot of lymphocyte membrane vesicles and fluorescent Megamix beads affirmed that the annexin V-positive vesicles were in the microparticle size gate (Fig. 2B). For quantification of the microparticle count, 5000 Trucount® beads were counted in a defined area and compared with the absolute number of microparticles in the microparticle gate. Non-stimulated lymphocytes showed a basic shedding of 4246 ± 1114 microparticles and this was significantly enhanced to 38,095 ± 9857 by the activation of T cells (*P* ≤ 0.001, Fig. 2C and D). The enrichment of the microparticle fraction could also be demonstrated by immunoblot analysis revealing the increased expression of LFA-1 (Fig. 2E). To exclude that any kind of cell death contributes to the Ca^2+^/A23187-induced release of microparticles we measured the amount of live and dead cells by trypan blue staining and also the release of LDH from T cells. As shown in Figure 2F and G there was no significant difference with regard to both parameters in the cells treated with and without Ca^2+^/A23187 stimulation.

### Analysis of proteasomes of microparticles shed by T lymphocytes

**Figure 3:**
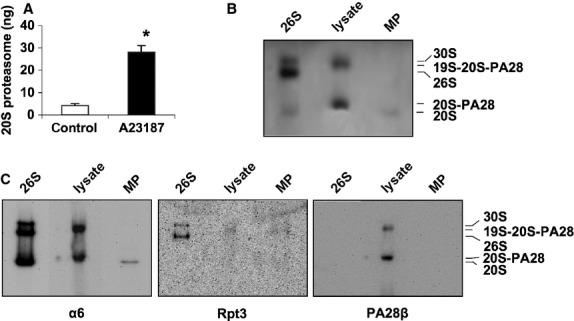
Quantification and analysis of proteasomes in microparticles. (A) Quantification of 20S proteasomes in the microparticle fraction (MP) by ELISA. T cells were stimulated by 2 μM A23187/1 mM CaCl_2_ (A23187) or left untreated (control). The MP fraction was isolated by ultracentrifugation and the MPs were disrupted by repeated freeze and thaw cycles. After precipitation of membrane residues 20S proteasomes were quantified by ELISA. Mean values ± SEM (*n* = 6) are shown and compared by the Mann–Whitney Rank Sum test, *P ≤ 0.002. (B and C) Native PAGE analysis of proteasome complexes in T cells and T cell MPs. Total lysate of T lymphocytes (lysate) and the MP fraction isolated by differential centrifugation from 2.5 × 10^8^ T cells stimulated for 45 min. with 1 mM CaCl_2_ and 2 μM A23187 were subjected to native PAGE. Human erythrocytes 26S proteasome (26S) was used as control containing 30S, 26S and 20S proteasome complexes, respectively. (B) After electrophoresis proteasome activity was visualized by overlay technique with 200 μM Bz-VGR-AMC as substrate. (C) Native immunoblot analysis was performed with antibodies to the subunits α6, Rpt3 and PA28β to detect the various proteasome complexes: 20S, 20S-PA28, 26S, 19S-20S-PA28, 30S.

To investigate whether the microparticles may be a source of extracellular proteasomes, we extracted their proteins by repeated freeze-thaw cycles. After centrifugation 20S proteasomes were detected in the supernatant fraction and quantified by ELISA (Fig. 3A). The microparticle fraction of resting 5 × 10^7^ lymphocytes contained 4 ± 1 ng 20S proteasomes, whereas the same amount of A23187/CaCl_2_ treated T cells contained 28 ± 3 ng 20S proteasome (corresponding to 4246 ± 1114 *versus* 38,095 ± 9857 microparticles measured in Fig. 2D). To study whether the extracellular proteasomes are proteolytically active native gel electrophoresis was performed to separate the different proteasome complexes of microparticles and of T cell lysates. As a control we used purified human erythrocyte 26S proteasome. Figure 3B shows the in-gel (substrate overlay technique) trypsin-like activity of proteasomal complexes after their separation by native gel electrophoresis. T cell lysates contain two proteolytically active complexes. As evidenced by immunoblotting with antibodies to subunit a6 (20S proteasome), to Rpt3 (19S regulator) and to PA28 (PA28β), the bigger of the two complexes contains the 19S regulator and PA28 in addition to the 20S proteasome. Thus, hybrid proteasome (19S-20S-PA28) is the predominant proteasome complex in T lymphocytes. The other proteolytically active complex in T cell lysates exhibits an electrophoretic mobility slightly slower than that of the 20S proteasome and represents 20S-PA28 proteasome complexes as shown by immune reactivity against the proteasome subunits α6 and PA28β. In contrast microparticles contain just proteolytically active 20S proteasome (Fig. 3C).

**Figure 4:**
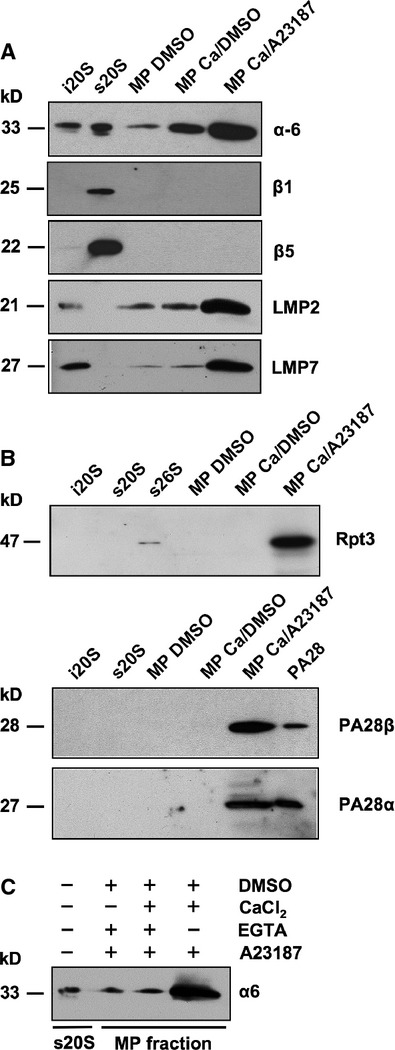
Detection of proteasomal subunits in microparticles. T lymphocyte microparticle fractions isolated from cell culture supernatants of control cells (MP DMSO), cells stimulated with 1 mM CaCl_2_ (MP Ca/DMSO) and cells treated with 1 mM CaCl_2_ and 2 μM A23187 (MP Ca/A23187) were subjected to SDS-PAGE and immunoblot analysis. Controls were human 20S immunoproteasomes (i20S), standard 20S (s20S) and 26S proteasomes (s26S) as well as PA28. (A) The MP fractions were analysed for proteasomal subunits α6, β1, β5, LMP2 and LMP7. (B) Proteins on immunoblots of controls and MPs were detected by antibodies for Rpt3 as well as PA28 α and β. (C) MP shedding was inhibited by depletion of Ca^2+^ with EGTA tracked by the detection of the α6 subunit.

Immunoblotting confirmed that proteasomes in microvesicles are immunoproteasomes containing the subunits β1i/LMP2 and β5i/LMP7. As reflected by an enhanced signal for β1i/LMP2, β5i/LMP7 and LFA-1 activation of T cells by the calcium ionophore A23187 and CaCl_2_ results in increased generation of microparticles (Figs 2E and 4A). On the other hand, inhibition of microparticle blebbing by EGTA resulted in a strong reduction of microparticle derived 20S proteasomes (Fig. 4C). Notably, the Ca/A23187 enhanced packaging of T cell 20S proteasomes into microparticles seems to go along with an export of proteasomal regulatory complexe subunits (Fig. 4B), which are not associated with the 20S core complex (compare Fig. 3B and C), because only under this experimental condition the 19S regulator subunit Rpt3 and PA28 were detectable.

### Microvesicles from human blood plasma contain proteasomes

Since proteasomes are released from cells by microvesicle shedding, we were interested to know whether proteasome-containing microvesicles can also be isolated from blood plasma of human volunteers. In preceding experiments we had found that the concentration of circulating proteasomes in plasma was higher when the plasma probes were stored frozen before the ELISA was carried out (239 ± 138 ng/ml frozen *versus* 148 ± 132 ng/ml unfrozen). The proteasome concentrations measured in frozen and unfrozen platelet free plasma were 140 ± 118 ng/ml *versus* 140 ± 114 ng/ml, respectively. Therefore, platelets were removed from the plasma of six healthy donors before microvesicles were isolated by centrifugation. As shown by immunoblotting microvesicles of all individuals contained 20S proteasomes (Fig. S1). Their mean concentration was 14.8 ± 3.8 ng 20S proteasome/ml plasma. Analysis of the remaining supernatant fraction revealed that in all samples proteasomes were also present in a vesicle-free form, the mean concentration of which was 164 ng/ml plasma (range 80–475 ng/ml). Thus, the ratio of microparticle bound to free 20S proteasome is about 1:11 suggesting that in plasma a mechanism must exist allowing the release of proteasomes from microvesicular structures.

### Microparticle breakdown by lipases releases free extracellular proteasomes

**Figure 5:**
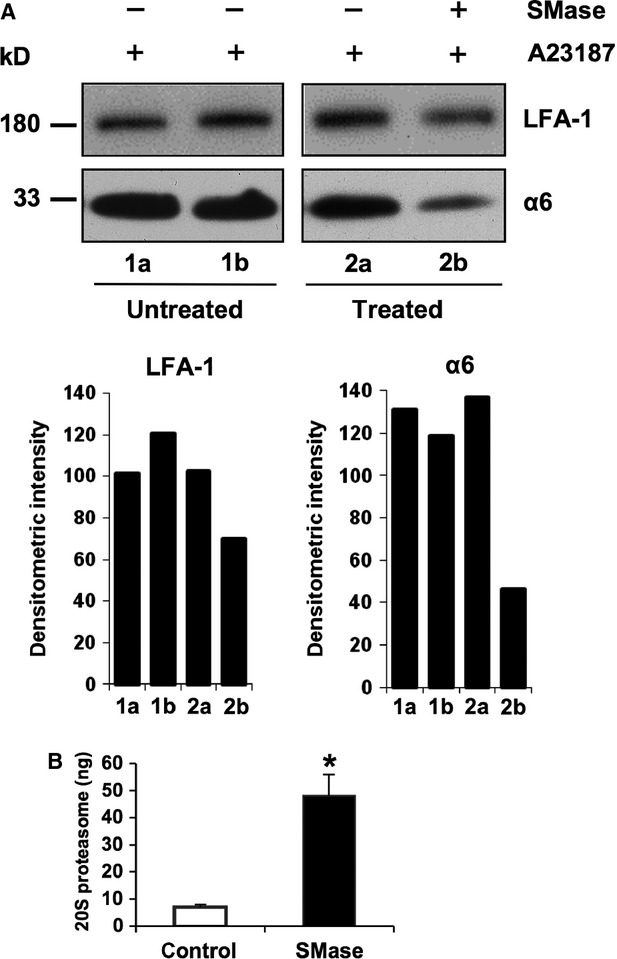
Turnover of microparticles with sphingomyelinase. (A) An aliquot of microparticles (MPs) from A23187-stimulated T lymphocytes, was used as a loading control for SDS-PAGE (lane 1a, 1b). A second aliquot was used for an *in vitro* degradation assay of the MPs by treatment (+) or non-treatment (−) with bacterial sphingomyelinase (SMase) at 37°C. The remaining lipid fraction was pelleted by centrifugation and dissolved by freeze-thaw cycles. Both membrane fractions were subjected to SDS-PAGE and analysed for their content of LFA-1 and proteasome subunit α6 (lane 2a, 2b). A quantitative evaluation of the band intensities was performed with Image J. (B) The supernatants of the microparticle SMase digestion as described in (A) were used for determination of liberated proteasomes. Mean values ± SEM (*n* = 4) are shown and the *P*-value were calculated by the Mann–Whitney Rank Sum test, *P = 0.029.

The levels of lipid-degrading enzymes such as secretory phospholipase A2 (sPLA2) and sphingomyelinase (SMase) are known to be increased in the blood plasma of patients suffering from several types of disorders 30–33. Fourcade and co-workers reported that the combined action of sPLA2 and SMase released free fatty acids from vesicle membranes 26. Therefore, we investigated whether of a commercially available SMase might hydrolyse microparticle membranes resulting in the release of proteasomes from microvesicles. Figure 5A shows that the amount of proteasome indicated by subunit α6 (detection of subunit LMP7 showed the same results; data not shown) and of the cell surface protein LFA-1 was significantly reduced in the vesicle fraction treated with SMase when compared to control samples. Accordingly, the proteasome content in the supernatant fractions of the SMase digestion samples was significantly increased and the liberation of proteasomes from vesicular structures by SMase appeared to be more efficient than repeated freeze-thaw cycles (compare Figs 5B and 3A). These data show that microparticles can be dismantled by sphingolipid hydrolysing enzymes resulting in the release of free proteasomes into the environment.

## Discussion

Since proteasomes were detected to circulate in blood plasma and their concentration is increased in patients with various malignancies, the question arose whether circulating proteasomes are released from damaged cells. Although initially a positive correlation between the elevated serum proteasome levels and a marker for cell death, like LDH, was observed in patients with malignant neoplasms and some autoimmune diseases 5–13 most other investigations in patients suffering from malignant and non-malignant diseases 11–36 found only poor correlations between markers of cell lysis and the level of circulating proteasomes. Therefore, processes other than cell disruption may contribute to the level of circulating proteasomes and we show here that T lymphocytes traffic proteasomes into their environment packed in microparticles (Figs 1D, 4A and B). This mechanism is used for basal as well as for enhanced outward transfer, since a rise in proteasome-containing microparticle release was observed in the presence of Ca^2+^ and a Ca-ionophore (Figs 2 and 4). Since many different stimuli for the activation of cells are responsible for microparticle release like the early phase of apoptosis, Ca^2+^-ions, phorbol esters, cytokines, complement factor C5a, C-reactive protein and high concentrations of ATP 16–37, it is not surprising that several diseases linked to a misguided immune response like SLE, polymyositis, primary Sjögren′s syndrome, sepsis and RA as well as hematologic malignancies and other forms of cancer go along with increased levels of serum microparticles and circulating proteasomes (Table S1).

Calculating the proteasome content in microparticles shed from cultured T cells (Figs 1 and 2) yields about 1 ng proteasome/1000–1300 microparticles. Since the number of microparticles in healthy individuals vary between 10^5^ and 10^6^/ml blood 38, a quantitative release of their content into plasma would result in a concentration of 10–1000 ng proteasome/ml plasma. Considering that plasma microparticles are thought to have various cellular origins including platelets, erythrocytes, leukocytes, endothelial cells and vascular smooth muscle cells 37, 400 ng circulating proteasomes/ml plasma as measured in normal individuals 15 fits well to our experimental findings.

Since circulating proteasomes in patients were directly quantified by ELISA technique, they must exist in a free, non-vesicular form, allowing binding of antibodies for reproducible quantification. Applying an *in-vitro* assay it was shown that two phosphodiesterases, cPLA2 and SMase, catalyse microparticle membrane turnover 26 and thus may release the vesicle content including proteasomes into body fluids. Our experiments using a commercially available bacterial SMase led to pronounced microparticle degradation and to liberation of vesicular proteasomes (Fig. 5) and thus sustain the likelihood of this mechanism to be physiologically relevant. Proinflammatory mediators foster the release of lipid-degrading enzymes, as sPLA2 was found to be released from rat calvarial bone-forming cells by IL-1 and TNF-α influence 39. Acid-SMase was reported to be secreted by endothelial cells by cytokine activation including interferon-γ and IL-1β 40, suggesting a pathophysiological relevance for these phospholipases. Elevated plasma levels of lipid-hydrolyzing enzymes have been linked to various disorders like rheumatoid arthritis (sPLA2) and sepsis (sPLA2, acid-SMase) 31,41 that are also characterized by increased levels of extracellular proteasomes 15. Since, the levels of microparticles were inversely correlated with sPLA2 activity 43, elevated levels of extracellular proteasomes are a reasonable consequence. Therefore, our *in vitro* experiments may indeed mimic the *in vivo* situation.

Our data are compatible with proteomic analyses of microparticles, which identified subunits of proteasomes and their regulators in platelet and T lymphocyte microvesicles 23–24. Moreover, our data show that proteasomes released from microparticles are proteolytically active. Whether proteasomes exhibit any proteolytic function in the extracellular environment remains unknown and is a matter of debate 14–15. Recently, Lai and colleagues detected that exosomes deriving from the endosomal compartment of mesenchymal stem cells also contain proteolytically active 20S proteasomes and they proposed that exosomal proteasomes may have a cardioprotective effect 44. On the other hand, microparticles as well as exosomes may be a potent source for autoantigens and provoke an immune response, because autoantibodies to proteasomal subunits were detected in the plasma of individuals with autoimmune disorders 45–46.

In summary, the data presented here provide a novel link between microparticle shedding and extracellular proteasomes and will require further investigations on microparticle count, plasma phospholipase activity and level of circulating proteasomes in healthy and diseased volunteers to evaluate the impact of this system on disease activity and prognostic value.

## Supporting information

**Figure S1** Analysis of vesicle-associated proteasomes in plasma of healthy individuals. **Table S1** Different pathophysiological states are associated with heightened levels of circulating proteasomes and mircoparticles.
