# Supplementary material for: T lymphocytes export proteasomes by way of microparticles: a possible mechanism for generation of extracellular proteasomes
Source: J Cell Mol Med. 2013 Oct 31;18(1):59–68. doi: 10.1111/jcmm.12160 (PMC3916118; doi:10.1111/jcmm.12160)
Supplement: Supplementary file 1 — Figure S1 Analysis of vesicle-associated proteasomes in plasma of healthy individuals. Table S1 Different pathophysiological states are associated with heightened levels of circulating proteasomes and mircoparticles. [file jcmm0018-0059-sd1.doc]

**Supplement Fig. 1** Analysis of vesicle-associated proteasomes in plasma of healthy individuals.

From blood of six healthy donors platelet-free plasma was obtained by differential centrifugation. Microvesicles from platelet-free plasma of six healthy donors isolated by ultracentrifugation (lane 1-6) were dissolved in RIPA buffer and tested by SDS-PAGE and immunoblotting for the presence of the proteasomal subunit α6. Immunoproteasome (i20S) was used as control.


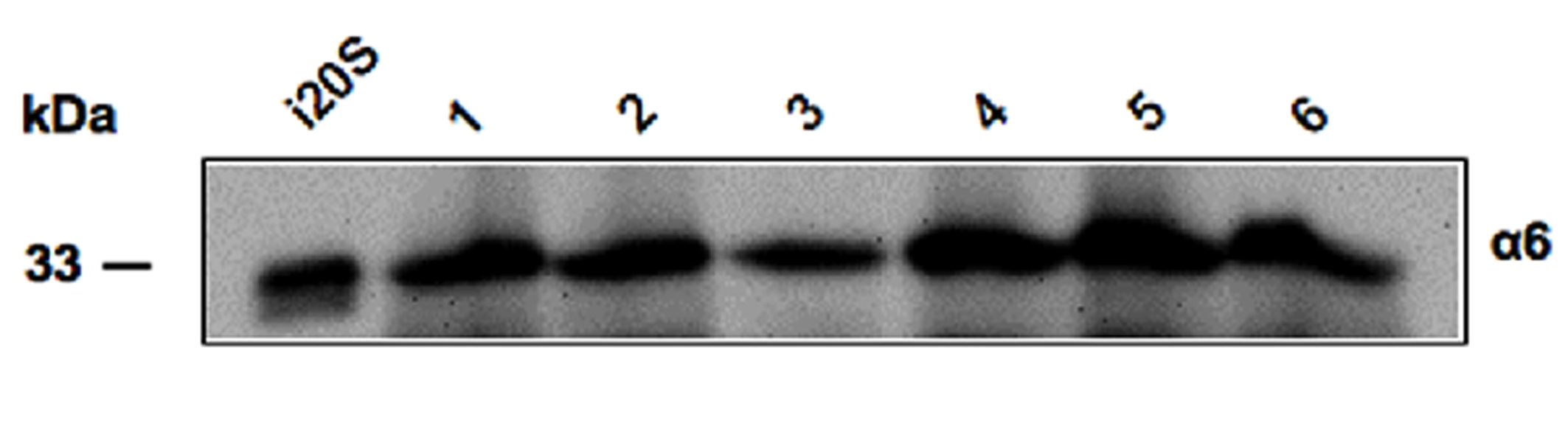


| **Supplement Table 1**  Different pathophysiological states are associated with heightened levels of circulating proteasomes and mircoparticles | | | | | |
| --- | --- | --- | --- | --- | --- |
|  |  |  |  |  |  |
|  |  | c proteasome |  | microparticles |  |
|  |  |  |  |  |  |
| acute respiratory distress syndrome | BAL | + | [1] | LMP, NeuMP | [2] |
|  | plasma | + |  | LMP,PMP |  |
| cancer | plasma | + | [3,4] | EMP,PMP | [5] |
| chronic hepatatis C | plasma | + | [3] | TMP | [6] |
| polymyositis | plasma | + | [7] | MMP, TMP, BMP, PMP | [8,9] |
| primary sjögren´s syndrome | plasma | + | [7] | tMP, PMP, LMP | [10] |
| rheumatoid arthritis | plasma | + | [7,11] | tMP, PMP | [10,12] |
| sepsis | plasma | + | [13] | tMP, PMP | [14] |
| systemic lupus erythematosus | plasma | + | [7] | tMP, PMP | [10] |
| trauma | blood | + | [13] | PMP, EMP | [15] |
|  |  |  |  |  |  |

microparticles (MP). LMP, lymphocyte MP; NeuMP, neutrophile MP; PMP, platelet-derived MP; EMP, endothelial MP; TMP, T lymphocytes MP; BMP, B lymphocytes MP; MMP, Monocytes MP; tMP, total MP (determined by different methods).

1. **Sixt SU, Adamzik M, Spyrka D, et al.** Alveolar extracellular 20S proteasome in patients with acute respiratory distress syndrome. *Am J Respir Crit Care Med*. 2009; 179: 1098-106.

2. **Guervilly C, Lacroix R, Forel JM, et al.** High levels of circulating leukocyte microparticles are associated with better outcome in acute respiratory distress syndrome. *Crit Care*. 2011; 15: R31.

3. **Wada M, Kosaka M, Saito S, et al.** Serum concentration and localization in tumor cells of proteasomes in patients with hematologic malignancy and their pathophysiologic significance. *J Lab Clin Med*. 1993; 121: 215-23.

4. **Lavabre-Bertrand T, Henry L, Carillo S, et al.** Plasma proteasome level is a potential marker in patients with solid tumors and hemopoietic malignancies. *Cancer*. 2001; 92: 2493-500.

5. **Campello E, Spiezia L, Radu CM, et al.** Endothelial, platelet, and tissue factor-bearing microparticles in cancer patients with and without venous thromboembolism. *Thromb Res*. 2011; 127: 473-7.

6. **Kornek M, Lynch M, Mehta SH, et al.** Circulating microparticles as disease-specific biomarkers of severity of inflammation in patients with hepatitis C or nonalcoholic steatohepatitis. *Gastroenterology*. 2012; 143: 448-58.

7. **Egerer K, Kuckelkorn U, Rudolph PE, et al.** Circulating proteasomes are markers of cell damage and immunologic activity in autoimmune diseases. *J Rheumatol*. 2002; 29: 2045-52.

8. **Baka Z, Senolt L, Vencovsky J, et al.** Increased serum concentration of immune cell derived microparticles in polymyositis/dermatomyositis. *Immunol Lett*. 2010; 128: 124-30.

9. **Shirafuji T, Hamaguchi H, Higuchi M, et al.** Measurement of platelet-derived microparticle levels using an enzyme-linked immunosorbent assay in polymyositis and dermatomyositis patients. *Muscle Nerve*. 2009; 39: 586-90.

10. **Sellam J, Proulle V, Jungel A, et al.** Increased levels of circulating microparticles in primary Sjogren's syndrome, systemic lupus erythematosus and rheumatoid arthritis and relation with disease activity. *Arthritis Res Ther*. 2009; 11: R156.

11. **Boilard E, Nigrovic PA, Larabee K, et al.** Platelets amplify inflammation in arthritis via collagen-dependent microparticle production. *Science*. 2010; 327: 580-3.

12. **Knijff-Dutmer EA, Koerts J, Nieuwland R, et al.** Elevated levels of platelet microparticles are associated with disease activity in rheumatoid arthritis. *Arthritis Rheum*. 2002; 46: 1498-503.

13. **Roth GA, Moser B, Krenn C, et al.** Heightened levels of circulating 20S proteasome in critically ill patients. *Eur J Clin Invest*. 2005; 35: 399-403.

14. **Tokes-Fuzesi M, Woth G, Ernyey B, et al.** Microparticles and acute renal dysfunction in septic patients. *J Crit Care*. 2012.

15. **Morel N, Morel O, Petit L, et al.** Generation of procoagulant microparticles in cerebrospinal fluid and peripheral blood after traumatic brain injury. *J Trauma*. 2008; 64: 698-704.
